# Exposure to a nanoplastic-enriched diet for fourteen days increases microglial immunoreactivity in the zebrafish telencephalon

**DOI:** 10.3389/fnmol.2025.1563086

**Published:** 2025-05-27

**Authors:** Robert A. Mans, Hannah Kelehear, Sarah Rotschafer, Clare Ganas, Brendan Uche-Moon, Gabrielle Call, Callie C. Mauersberg, Justin Toller, Andrew Diamanduros

**Affiliations:** ^1^Department of Biology, College of Science and Mathematics, Georgia Southern University–Armstrong Campus, Savannah, GA, United States; ^2^Department of Biomedical Sciences, School of Medicine, Mercer University, Savannah, GA, United States; ^3^Department of Biology, College of Science and Mathematics, Georgia Southern University, Statesboro, GA, United States

**Keywords:** nanoplastics, astrocytes, gliosis, zebrafish, microglia, neuroinflammation, 4C4, GFAP

## Abstract

Microscopic plastic particles (micro- and nanoplastics) are an emerging environmental contaminant detected in air, soil, water, and human food supplies. Experiments using zebrafish have shown that polystyrene nanoplastics will infiltrate numerous organ systems after ingestion, including the brain, liver, muscle, and reproductive organs. Additionally, work in rodent models and cell culture has demonstrated that nanoplastics can induce inflammatory responses by microglia and alter astrocyte function. However, the responses of microglia and astrocytes in the zebrafish brain caused by daily exposures to nanoplastics have not been tested previously. In the current study, adult zebrafish were exposed to a nanoplastic-enriched diet consisting of *Artemia* brine shrimp containing 44 nm polystyrene spheres, and reactive gliosis by microglia and astrocytes was examined. Microglial 4C4-immunoreactive protein was elevated in the brains of zebrafish exposed to the nanoplastic-enriched diet. Levels of glial fibrillary acidic protein (GFAP) were not affected by plastic exposure. It was determined that microglial, but not astrocytic, markers were elevated in the zebrafish brain after 14-days of exposure to a nanopolystyrene-enriched diet. These findings contribute to our understanding of how a pervasive environmental contaminant, nanoplastics, may impair brain health, especially during the initial stages of nanoplastic exposure. Additionally, this is the first study using zebrafish to evaluate glial activation in the context of nanoplastic-contaminated foods.

## Introduction

It is now widely recognized that plastic pollution, particularly within oceans, is pervasive (Derraik, 2002). Approximately eight million tons of plastic enter oceans annually (Jambeck et al., 2015), and the rate of plastic deposition is projected to increase (Geyer et al., 2017). The pathways for plastic input into oceans are diverse. Plastic sources include illegal dumping activities, riverine (Lebreton et al., 2017) and atmospheric transport (Allen et al., 2019) from coastal and inland areas, direct at-sea littering from shipping, fishing and aquaculture (GESAMP, 2021; Jambeck et al., 2015), and wastewater from washing clothes (GESAMP, 2021). The most abundant plastic polymers detected in the Atlantic Ocean are polyethylene, polypropylene, and polystyrene (Pabortsava and Lampitt, 2020). Due to the gradual breakdown of plastics in the environment, a continuum of plastic particle sizes exists within oceans. Particle sizes have been categorized as macro- (greater than or equal to 1 cm), meso- (1–10 mm), micro- (1–1,000 μm), and nanoplastics (1–1,000 nm) (Hartmann et al., 2019). The smallest particles (micro- and nanoplastics) have received considerable attention in aquatic research, because the smallest plastics are not only most abundant in ocean water, but they are also the most likely to be internalized by marine life (Lenz et al., 2016). Plastics have been detected inside organisms at every trophic level of aquatic food webs, including zooplankton (Mattsson et al., 2017), mussels (Avio et al., 2015), crabs (Yu et al., 2018), fish (Baechler et al., 2020), mammals (Nelms et al., 2018) and birds (Hammer et al., 2016). Furthermore, microplastics can be transferred from the reproductive organs of fish into eggs and developing larvae (Pitt et al., 2018). The pervasive nature of plastic pollution and its propensity to be propagated through food chains invokes the possibility that commercially important fish and shellfish will experience extensive accumulation of plastics due to ingestion over a lifetime. Barboza et al. (2020) conducted a survey of 50 commercially important fish species in the northeast Atlantic Ocean, and it was determined that 49% of sampled fish had internalized microplastics and exhibited evidence of neurotoxicity. Therefore, humans that consume fish and shellfish are likely consuming small quantities of micro- and nanoplastics chronically over a lifetime (Cox et al., 2019). Additionally, microplastics can enter other human food sources through numerous steps of the supply chain which include agriculture, processing facilities and packaging (Lin et al., 2022). In an agricultural setting, plants are capable of internalizing microplastics derived from the soil and rainwater (Azeem et al., 2021). Additionally, several types of plastics are used in components for equipment involved in food processing, food packaging and water purification (Lin et al., 2022; Weisser et al., 2021). Therefore, interactions between food, equipment and packaging are a significant plastic source. Plastics have been detected within honey, sugar, water, alcohol, table salt, canned fish and other packaged meats (Cox et al., 2019; Lin et al., 2022; Qian et al., 2021). Using the most sensitive detection method to date, which incorporated nano-sized detection not possible in previous studies, Qian et al. (2021) detected micro- and nano-sized plastics within several brands of commercially available bottled water. On average, 240,000 micro/nanoplastics were detected per liter of bottled water. Importantly, 90% of the plastics were in the nanometer size range, and the vast majority of the nanoparticles were comprised of polystyrene. Since the limit of detection did not permit detection below 100 nm, and some particle types were not specifically identified, the authors predict that the number of micro-nanoparticles may be as high as 1 million per liter. Aside from internalization from food sources, it is likely that internalization of airborne plastics occurs due to inhalation, as microplastics are distributed within building air conditioning systems (Cox et al., 2019). Due to chronic exposure, the potential for micro-/nanoplastics to enter the bloodstream and penetrate organs is alarmingly high. In fact, microplastics have been detected in human blood (Leslie et al., 2022), breast milk (Ragusa et al., 2022), liver (Horvatits et al., 2022), testes (Zhao et al., 2023), and brain (Campen et al., 2024). However, the short- and long-term effects of nanoplastics penetrating tissues, especially the brain, are largely unknown.

An increasing number of research groups have begun to investigate neurobiological outcomes of micro/nanoplastic exposures. It was first demonstrated in fish research models—Crucian carp (Mattsson et al., 2017) and zebrafish (Sarasamma et al., 2020)—that nano-sized polystyrene particles delivered through the food chain can penetrate the blood-brain barrier (Mattsson et al., 2017; Sarasamma et al., 2020). Plastic particles were found in neural tissue (Mattsson et al., 2017; Sarasamma et al., 2020), and this was associated with altered brain morphology (Mattsson et al., 2017) and declining neurotransmitter volumes (Sarasamma et al., 2020) in these fish models. In rodents, upregulation of brain inflammatory gene expression (Gaspar et al., 2023; Liang et al., 2022), deficits in brain mitochondrial energy production (Liang et al., 2022), loss of dopaminergic neurons (Mattsson et al., 2017) and increased blood-brain barrier permeability (Liang et al., 2022; Shan et al., 2022) have been observed after daily polystyrene ingestion. Deficits in learning and memory have also been observed in mice after consuming nanoplastics for seven weeks (Paing et al., 2024). The aforementioned studies entailed exposure periods of seven days (Shan et al., 2022), 21 days (Gaspar et al., 2023), 28–30 days (Liang et al., 2022; Sarasamma et al., 2020), 49 days (Paing et al., 2024), or 64 days (Mattsson et al., 2017). More long-term exposure (180 days) to brain-permeable polystyrene particles via drinking water caused reduced spine density, reduced synaptic proteins, and ultrastructural changes in the blood-brain barrier in the hippocampus of mice (Jin et al., 2022). In sum, these studies clearly indicate that daily polystyrene micro-nanoparticle ingestion may lead to detrimental changes in brain health. However, the characteristics of brain pathologies that may be caused by daily nanoplastic exposures are poorly understood, especially in the context of nanoplastic-contaminated foods.

Inflammatory immune responses contribute to the etiology of several nervous system pathologies including Alzheimer’s disease (Kinney et al., 2018), Parkinson’s disease (Rojo et al., 2020), multiple sclerosis (Haase and Linker, 2021), spinal cord injury (Hellenbrand et al., 2021) and stroke (Jayaraj et al., 2019). In the mammalian brain, a resident population of immuno-competent cells—microglia and astrocytes—are responsible for combating pathogens and other insults in the brain and spinal cord (Matejuk and Ransohoff, 2023). As the innate immune cells of the CNS, microglia patrol the CNS, removing dead cells, cellular debris (Damisah et al., 2020) and toxic macromolecules from the brain interstitium (Neumann et al., 2009). A blend of microglial phenotypes, ranging from homeostatic (M2) to inflammatory (M1) are common. When microglia encounter CNS injury, infection, harmful brain-derived macromolecules or harmful man-made xenobiotics, they transition from a ramified, homeostatic phenotype (M2) to a more reactive (M1) phenotype (Liddelow et al., 2017). In the reactive state, microglia undergo a distinct morphological transformation, assuming an amoeboid shape with truncated processes (Jonas et al., 2012). Additionally, reactive microglia secrete the cytokines interleukin 1-alpha (IL-1α), tumor necrosis factor alpha (TNF-alpha), and C1q (Matejuk and Ransohoff, 2023). Cytokines secreted by microglia recruit neighboring microglia to the site of insult (Matejuk and Ransohoff, 2023). Importantly, the reactive phenotype serves a homeostatic function—toxic proteins, damaged cells and other debris are cleared from the brain by phagocytic microglia. In fact, a blend of M2 and M1 characteristics are typically observed in microglia. However, excessive M1 activity, a hyperinflammatory state, can lead to damage to the CNS (Muzio et al., 2023). For example, hyperreactive microglia are known to aberrantly prune healthy synapses (Hong et al., 2016). Additionally, microglial cytokines recruit astrocytes to assume a reactive phenotype, which exacerbates the inflammatory state of the CNS after an insult or injury (Matejuk and Ransohoff, 2023).

The prospect of microglial activation in response to nanoplastic exposure has been investigated in culture systems and rodents. In cell culture, microglia have been shown to phagocytose nano-sized polystyrene spheres, releasing cytokines into culture media (Paing et al., 2024). In mice, reactive microglial morphology and inflammatory cytokine release have been observed after oral intake of polystyrene particles (Shan et al., 2022; Yang et al., 2023). However, microglial activation in response to nanoplastics in the zebrafish model system is lacking in the published literature. Filling this gap in the literature would inform the use of zebrafish for studying neurological impacts of nanoplastic exposures from both a biomedical and ecological perspective.

Astrocytes perform numerous functions to support homeostasis and communication within neural networks in the mammalian CNS, and they protect the brain in the case of insult, injury and disease (Sarasamma et al., 2020). The zebrafish model system contains a population of astrocyte-like cells, which have been termed astroglia, that arise from a robust population of radial glial cells (Baumgart et al., 2012; Kim et al., 2022; Wang et al., 2019). In the case of an injury, disease, infection, or other insult, a subpopulation of astrocytes will assume a reactive (gliotic) phenotype to contain the threat to CNS health (Senathirajah et al., 2021). During the process of astrogliosis, reactive astrocytes commonly exhibit several functional changes, which include secretion of inflammatory cytokines (tumor necrosis-alpha (TNF-alpha) and interleukin-6 (IL-6)), migration to the site of injury, hypertrophy of processes, and phagocytosis (Damisah et al., 2020). Also, the transition to the reactive phenotype is often accompanied by increased expression of the cytoskeleton-associated glial fibrillary acidic protein (GFAP). For example, in the zebrafish brain, an increase in GFAP occurs following injury to the brain [olfactory bulb (Wang et al., 2019); telencephalon (Baumgart et al., 2012) and in a genetic model of scoliosis (Walpole et al., 2012)].

**FIGURE 1:**
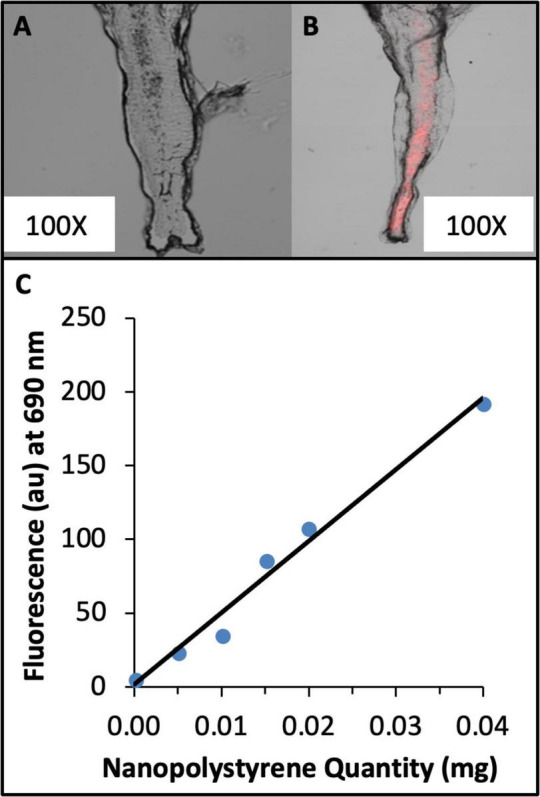
Red fluorescent polystyrene nanoparticles (nPS) were internalized by *Artemia* brine shrimp during a 24-h incubation in nPS-contaminated saltwater. **(A)** *Artemia* incubated in non-contaminated saltwater. **(B)** *Artemia* incubated in nPS-contaminated saltwater. **(C)** Standard curve of red fluorescence from homogenized shrimp samples spiked with nanoplastics. Nanoplastic quantities (mg): 0, 0.005, 0.010, 0.015, 0.020, 0.040. *N* = 3 samples per quantity. *R*^2^ = 0.9898.

A wide range of CNS conditions exhibit elevated numbers of reactive astrocytes, and GFAP levels are proportional to the extent of CNS pathology (Capone et al., 2021; Rovira et al., 2023). Therefore, exposure to environmental xenobiotics, such as nanoplastics, that induce inflammatory crosstalk between microglia and astrocytes may increase the susceptibility of the human population to neurodegenerative conditions (Figure 1). However, understanding of astrocytic responses to nanoplastic exposure is very limited. *In vitro*, high concentrations of nanoplastics will induce inflammation-linked changes in astrocyte gene expression (Kwon, 2022). Conversely, *in vivo*, mice exhibit a decline in GFAP-expression after ingesting micro-nanoplastic-enriched water for three weeks (Gaspar et al., 2023). No studies testing astrocyte responses to nanoplastics in zebrafish have been published. This significant knowledge gap regarding the responses of astrocytes to nanoplastics should be remedied to fully understand the toxic effects of nanoplastics on brain physiology in both short- and long-term exposures.

We sought to evaluate microglial and astrocytic responses to polystyrene nanoparticle consumption using zebrafish as a model organism. To this end, we tested a trophic nanoplastic transfer protocol using polystyrene-enriched *Artemia* brine shrimp as a daily food source for adult zebrafish followed by biochemical analysis of homogenized zebrafish brain tissue and laser scanning confocal microscopy of zebrafish brains.

## Results

### Production of a nanoplastic-contaminated food source

We sought to generate a plastic-contaminated food source meeting several requirements: (1) the size and type of plastic should match the size and type that are commonly encountered in the environment and in human food supplies, (2) fish should consume the entire quantity of food, (3) the amount of plastic should be quantifiable, (4) the process of food production should be highly reproducible. To this end, we chose live brine shrimp (*Artemia*), a food source that zebrafish enthusiastically pursue. To enrich live shrimp with nanoplastics, we took advantage of the property that they are non-specific filter feeders that have been shown by other groups to internalize microplastics suspended in water (Wang et al., 2019). Forty-four nm polystyrene nanospheres labeled with a red fluorescent dye that could be visualized and quantified in a spectrophotometer were chosen as a plastic source. As Qian et al. (2021) and others have shown, nano-sized plastics are by far the most abundant size of microplastic detected in the environment, and polystyrene is one of the most common plastics used by humans. Additionally, polystyrene was the most abundant nano-sized plastic type detected in commercially available bottled water (Qian et al., 2021). According to other studies using brine shrimp as an aquatic food source (Kim et al., 2022), 1000 shrimp is the appropriate daily allocation for an adult zebrafish. Therefore, batches of 1000 shrimp were incubated in glass beakers containing oxygenated artificial seawater (ASW, 10 parts per thousand) supplemented with nanoplastics at a concentration of 0.15 g polystyrene/50 mL ASW overnight. After overnight exposure to nanoplastics, *Artemia* were collected in a sieve, rinsed with deionized water for 60 s to remove plastic adhering to the outside of the shrimp, suspended in fresh water and then collected. To visually confirm that the brine shrimp were internalizing nanoplastics, a small sample of *Artemia* were drop-fixed in 4% paraformaldehyde, fixed to slides, and imaged with a scanning fluorescent confocal microscope using a 660 nm excitation/690 emmission filter. Red fluorescent particles were clearly observed within the digestive tracts of nPS-exposed shrimp but not within the non-exposed controls (Figures 1A, B).

Having confirmed nanoplastic internalization by the Artemia, we sought to estimate the mass of nanoplastics (nPS) within a sample of 1000 plastic-exposed shrimp. Shrimp were homogenized in phosphate-buffered saline supplemented with a Tween-20 detergent. The fluorescence intensity of shrimp homogenates was measured in triplicate from nPS- and non-nPS-exposed Artemia using a spectrophotometer. These fluorescence values were fitted to a standard curve generated from Artemia homogenates supplemented with nanoplastic solution (Figure 1C). The average quantity (+/– SE) of nPS in the brine shrimp after incubation in nPS-contaminated salt water for approximately twenty-four hours was calculated to be 2.53 +/− 0.81 micrograms nPS per 1000 shrimp (*N* = 8). Therefore, it was concluded that each zebrafish would consume approximately 2.5 micrograms of nanopolystyrene each day. It has been estimated that humans may consume 0.1 grams–5.0 grams of plastics weekly (Senathirajah et al., 2021). If this dosage is allometrically scaled to adult zebrafish [assuming an average human mass of 62 kg (Walpole et al., 2012) and a zebrafish mass of 0.5 grams], a proportional daily plastic dosage in zebrafish should fall in the range of 0.1 micrograms–5.8 micrograms. Since the calculated exposure in the current study equals 2.53 +/− 0.81 micrograms of nPS fed to zebrafish daily, we concluded that the plastic exposures in the current study were within the range of exposures estimated to be experienced by human adults.

### Microglial and astrocytic immunoreactivity in brain homogenates

**FIGURE 2:**
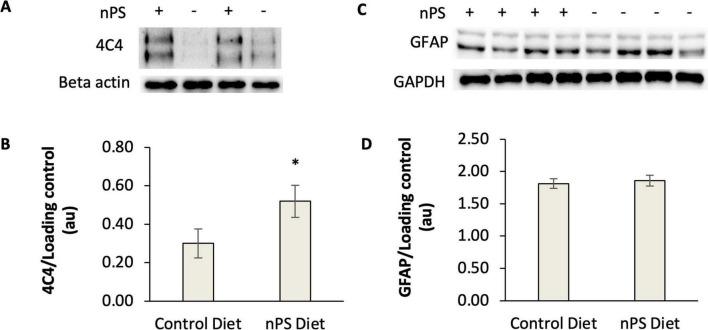
Western blot analysis of 4C4-immunoreactive proteins and GFAP in the telencephalon of adult zebrafish fed a nanoplastic-contaminated diet for fourteen days. The symbols “+” or “−” indicate presence or absence, respectively, of nanoplastics (nPS) in the diet. **(A)** Example western blots for 4C4 antibody. Beta actin is the loading control. **(B)** Quantification of 4C4 western blots from nPS-exposed fish (*N* = 12 fish) and controls (*N* = 11 fish). *P < 0.05, *t*-test. Hedges *g* = 0.81. **(C)** Example western blot for GFAP. GAPDH is the loading control. **(D)** Quantification of GFAP western blots from nPS-exposed fish (*N* = 14 fish) or controls (*N* = 14 fish) for 4C4. *P* = 0.46, *t*-test. Error bars represent standard error.

Next, we sought to determine if hallmark features of reactive gliosis—elevated microglial and astrocytic immunoreactivity—increased after daily exposure to nanoplastic-contaminated food. To this end, several cohorts of male and female adult zebrafish were fed a suspension of plastic-enriched *Artemia* brine shrimp once daily for 14 consecutive days. The 14-day timepoint was predicted to be sufficient to induce reactive gliosis, and it is relatively unexplored in the literature. At the conclusion of the feeding protocol, markers for microglia and astrocytes were measured in the telencephalon region of the zebrafish brain. The telencephalon was evaluated due to the presence of several key functional areas that are equivalent to the human cortex, hippocampus, amygdala and striatum. To quantify microglia, we employed the 4C4 primary antibody, which labels galectin-3 binding protein in macrophages and is well established for labeling microglia in fish models (Rovira et al., 2023). Consistent with other studies in zebrafish (Rovira et al., 2023), a western blot of homogenized brain samples revealed a mixture of 100–200 kDa proteins with immunoreactivity (ir) to the 4C4 antibody (Figure 2A). 4C4-ir (normalized to the loading control) was elevated in the brains of zebrafish that consumed nanoplastic-enriched *Artemia* relative to controls, suggesting that microglial activation had increased in the telencephalon after nanoplastic exposure. The mean ratio of 4C4/loading control (+/− SE) was 0.52 +/− 0.08 in nPS-exposed fish (*N* = 12 fish) compared to 0.30 +/− 0.07 in the control group (*N* = 11 fish); *P* = 0.03, *g* = 0.81 *t* = 1.87, *df* = 21, one-tailed *t*-test (Figures 2A, B).

In parallel with our measurements of microglial immunoreactivity, we conducted experiments to explore if astrocytes were transitioning to a reactive phenotype as a consequence of a plastic-contaminated diet. It was expected that GFAP levels would increase if greater numbers of astrocytes were exhibiting a reactive phenotype. After a 14-day plastic-contaminated diet, GFAP remained unchanged in the telencephalon (*P* = 0.46, *t*-test). The mean GFAP/GAPDH ratio (+/−SE) was 1.86 +/− 0.36 in nPS-exposed fish (*N* = 14 fish) compared to 1.81 +/− 0.34 in the control group (*N* = 14 fish) (Figures 2C, D). These results suggest that astrocytes were not being recruited to reactive cascades as rapidly as microglia after a 14-day exposure to a nanoplastic-contaminated diet.

### Microglial immunostaining

**FIGURE 3:**
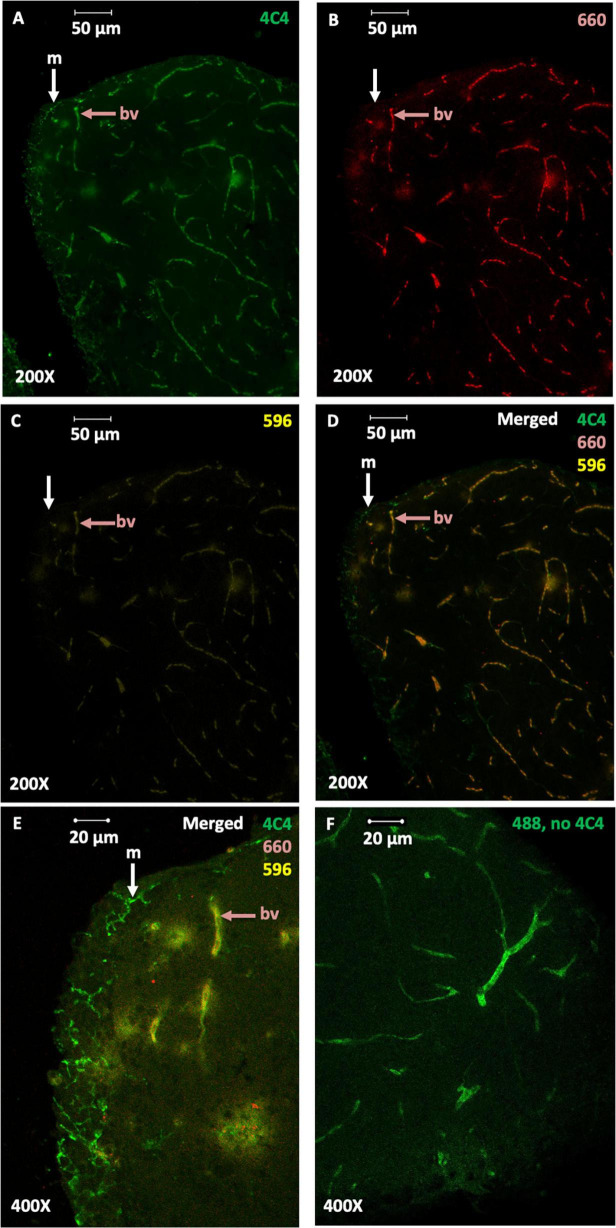
4C4 immunoreactivity and blood vessel autofluorescence in the telencephalon. The mediodorsal aspect of one telencephalon is shown. Images represent maximum projections from ten confocal scans taken under 200× or 400× magnification. **(A)** 4C4 immunoreactivity visible with 488 nm excitation. Arrow “m,” white, indicates microglia. Arrow “bv,” red, indicates a blood vessel. Autofluorescence was induced in blood vessels “bv,” but not microglia by excitation at the 660 nm **(B)** and 596 nm **(C)** wavelengths. **(D,E)** The merged images clearly differentiate green 4C4 immunoreactive microglia (“m”) from blood vessels (“bv”), which appeared yellow. Red punctate objects are also visible at the 660 nm wavelength exclusively. **(F)** Autofluorescence of blood vessels (“bv,” red arrow) induced with a 488 nm laser in the absence of 4C4 primary antibody.

**FIGURE 4:**
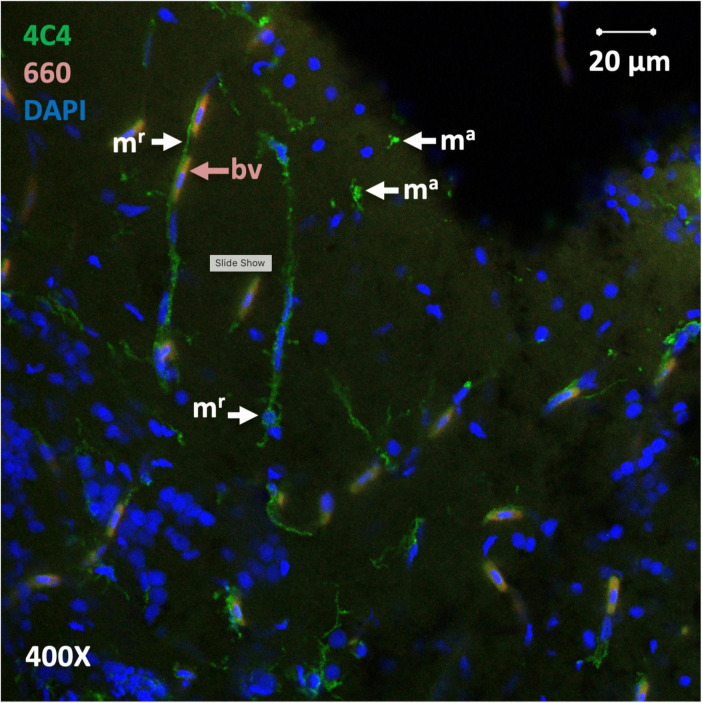
Microglial morphologies in the telencephalon. Maximum projection from ten confocal scans in the telencephalon at 400× magnification. Image was generated from merged images of microglial 4C4 immunoreactivity (green) and blood vessel autofluorescence induced by excitation at 448 nm and 660 nm wavelengths. Merged images enabled differentiation between yellow blood vessels (“bv” at red arrow) and microglia. Nuclei are counterstained with DAPI (blue). Ramified microglia (“m^r^”) and more amoeboid microglia (“m^a^”) are visible.

**FIGURE 5:**
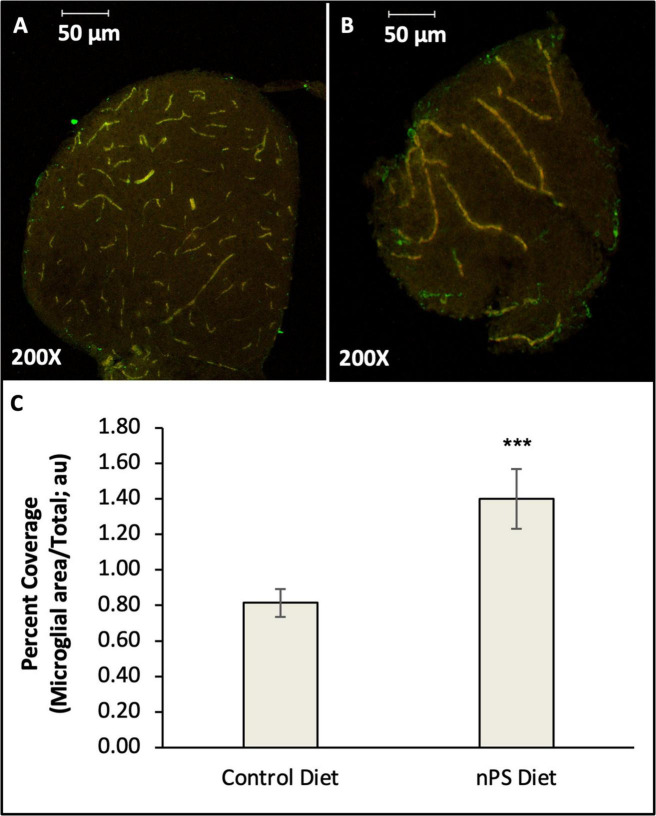
Density of reactive microglia in the telencephalon of adult zebrafish fed a nanoplastic-contaminated diet or control diet for fourteen days. Slices were immunostained with 4C4 primary antibody (green) and imaged with a scanning confocal microscope. Yellow cells are autofluorescent blood vessels excited with 660 nm and 594 nm lasers. **(A)** Control brain. **(B)** Brain from a fish exposed to a nanoplastic (nPS)-enriched diet. **(C)** Mean density of 4C4-ir microglia expressed as a percentage of total slice area. *N* = 64 slices from 7 brains for Control diet. *N* = 68 slices from 7 brains for nPS diet. ***P = 0.001 (Student’s *t*-test).

Next, we used an imaging approach to further evaluate the density of 4C4-ir microglia in the brains of zebrafish exposed to nanoplastic-enriched diet. A cohort of fish were fed a nanoplastic-contaminated diet or control diet for 14 days, then, brains were removed, fixed in 4% paraformaldehyde, sectioned, and immuno-stained with the 4C4 primary antibody to label microglia. We expected to see an increase in the density of microglia in the brains of fish exposed to a 14-day plastic-contaminated diet relative to fish on the control diet. Using a scanning laser confocal microscope, numerous cells with the size and shape consistent with microglia were detected using a 488 nm excitation laser, especially in the gray matter concentrated at the edges of the slices (Figures 3E, 4, 5). Several microglial morphologies were evident, ranging from highly ramified (Figure 3E, arrow *m*; Figure 4, arrow *m^r^*) to more amoeboid with retracted processes (Figure 4, arrow *m^a^*). In some cases, microglial processes were abutted against blood vessels (Figure 4). Robust green fluorescence was also observed in blood vessels excited by a 488 nm laser (Figure 3A, arrow *bv*; Figure 3F, arrow “bv”). However, negative-control slices not exposed to the 4C4 primary antibody exhibited the same pattern of blood vessel fluorescence (Figure 3F), indicating blood vessels were autofluorescent and not specifically immunolabeled with the 4C4 antibody.

In order to quantify the microglia within each slice, it was necessary to differentiate the vascular endothelial cells from microglia. To specifically identify blood vessels, the autofluorescent properties of blood vessels in the red and orange light spectra were utilized. Excitation at two wavelengths in the red and orange spectra (660 nm and 596 nm, respectively) selectively induced red and yellow fluorescence in the blood vessels (Figures 3B, C, arrow bv). By merging these images with the images from 488 nm excitation (green), the blood vessels exhibited a bright yellow wavelength reliably distinguishable from the green fluorescence of 4C4-ir microglia (Figures 3D, E). Next, the percent coverage of green 4C4-ir cells and processes, excluding blood vessels, within each slice was measured using Image J measurement tools. Consistent with the 4C4 western blots, the telencephalon of fish exposed to a nanoplastic-enriched diet exhibited increased density of 4C4-ir microglia relative to controls. The average percent coverage (+/− SE) in the slices from plastic-exposed fish was 1.40 +/− 0.17% (*N* = 68 slices from 7 fish) compared to 0.81 +/− 0.08% in controls (*N* = 64 slices from 7 fish). *P* = 0.001, *g* = 0.54, *t* = 3.11, df = 130, one-tailed *t*-test (Figure 5).

### Detection of nanoplastics within zebrafish brains

Next, we sought to determine the relationship between the glial measures in the telencephalon with the amount of polystyrene nanoplastics that had penetrated the zebrafish brain. Previous studies have shown that fluorescent microplastics can be measured in homogenized tissue using spectrophotometry. Therefore, the olfactory bulbs were collected from a sub-set of brains in which glial markers (4C4 and GFAP) had been measured in the telencephalon. Olfactory bulbs were homogenized, and fluorescence was measured using a 660 nm excitation/690 emission filter that matched the profile of the nanoplastics used in this study. The fluorescence intensity in the olfactory bulbs from fish on a nanoplastic-enriched diet (*N* = 4 fish) was indistinguishable from the fish on a control diet (*N* = 4 fish). Notably, the control tissue exhibited a background fluorescence in the red wavelength. This background fluorescence may have precluded detection of nanoplastics in the fish on the plastic-enriched diet. As an alternative approach, the brain slices assayed for microglial density were examined for instances of red fluorescent nanoplastics using the same excitation/emission profile. Instances of red punctate fluorescence consistent with the size and shape of a 44 nm particle were observed in the brains of the plastic-fed fish (Figures 3D, E). However, instances of red autofluorescence of various shapes and sizes were detected from unknown sources within the tissue. The presence of these artifacts prevented a specific, objective measure of plastic instances in the tissue from fish on the plastic-enriched diet. Therefore, the presence of nanoplastics cannot be ruled out, but attempts to measure nanoplastics within the brain tissue were inconclusive overall.

## Discussion

In the current study, we evaluated the effects of polystyrene nanoparticle consumption on microglial and astrocytic markers for reactive gliosis using adult zebrafish as a model organism. To this end, zebrafish were fed nanoplastic-enriched brine shrimp once daily for fourteen days, and the telencephalon—a cognitive center of the zebrafish brain—was evaluated for increased immunoreactivity of microglial and astrocytic markers associated with reactive phenotypes. This is the first study evaluating microglia and astrocyte responses in the adult zebrafish brain in the context of nanoplastic exposures. Consistent with studies in rodents and cultured microglia, we find that exposure to nanoplastics increases microglial immunoreactivity in the zebrafish brain. Specifically, the density of 4C4-immunoreactive microglia–a common measure of microglia in zebrafish–was elevated in zebrafish exposed to a plastic-enriched diet compared to fish on a control diet. An evaluation of microglial morphologies in the reactive versus resting state can yield important insights. However, the overwhelming density of fine 4C4-ir processes mixed with cell bodies precluded our ability to reliably count microglia of different morphologies. In agreement with our observation of increased microglial immunoreactivity after nanoplastic exposure, the level of total 4C4-ir protein, as measured by western blotting, was elevated in the telencephalon in a separate cohort of fish exposed to a plastic-enriched diet compared to controls.

Galectin-3 binding protein (LGALS3BP), the established target of the 4C4 primary antibody, is expressed broadly by macrophages and may be secreted or carry out its functions intracellularly (Capone et al., 2021). Notably, the innate immune system of the brain includes non-microglial immune cells such as bone marrow-derived T-cells that reside in the meninges (Kwon, 2022). Therefore, it is unknown if non-microglial immune cells were labeled in concert with microglia during imaging studies. Additionally, the source of 4C4 immunoreactive protein observed during western blotting likely included contributions from non-microglial cells in the brain. Still, the observed elevation of 4C4 immunoreactivity is broadly consistent with a reactive response of microglia to injury, as 4C4-ir microglia are known to become elevated in the zebrafish brain after tissue ablation (Baumgart et al., 2012; Var and Byrd-Jacobs, 2019).

As an additional measure of reactive gliosis, we measured GFAP, a marker for astrocyte activation that is commonly associated with neuroinflammation, in the same samples evaluated with 4C4. This is the first examination of astrocytes in the zebrafish brain after exposure to nanoplastics. In contrast to the response of the microglial marker, levels of GFAP were found to be unchanged in the zebrafish telencephalon after exposure to a nanoplastic-contaminated diet. These results suggest that consuming nanoplastics at the current dosage for fourteen days is insufficient to cause reactive gliosis in astrocytes in zebrafish. In the context of other studies, subjecting astrocytes to a seven-day nanoplastic exposure in culture causes an increase in inflammatory gene expression (Marcellus et al., 2024). However, the direct dosage of nanoplastics to astrocytes in culture likely caused exposure levels many folds higher than the current *in vivo* feeding study. A higher dosage of plastics, or a treatment of longer duration may be required to cause a change in GFAP in zebrafish. In mice administered nanoplastic-contaminated water for thirty days, a decline in brain GFAP was observed (Gaspar et al., 2023). As with other studies using drinking water or immersion to expose animals to nanoplastics, the quantity of plastics consumed daily by the animals in the study by Gaspar et al. (2023) cannot be determined. Therefore, a comparison of plastic quantity between Gaspar et al. (2023) and the current study cannot be achieved. It remains to be determined if the longer dosage used by Gaspar et al. (2023) will cause a similar decline of GFAP in zebrafish.

To best mimic human exposures to dietary nanoplastics, a conservative exposure frequency and dosage were implemented in the current study. We predicted that this exposure regime would be more relevant to human populations than an immersion protocol in which the zebrafish home tank water is contaminated with nanoplastics. Indeed, the calculated mass of plastic fed to zebrafish once daily (approximately 2.5 micrograms) was found to be biologically relevant when allometrically scaled to human exposure estimates [0.1–5.8 micrograms (Senathirajah et al., 2021)]. We find this methodology to be a strength of the current study relative to other *in vivo* procedures.

The current study was limited in that nanoplastics could not be conclusively documented within the zebrafish brain after exposure to a nanoplastic-contaminated diet. Two approaches—fluorescent spectrophotometry and fluorescent microscopy—were insufficient to detect plastics above background. The proclivity of biological molecules and non-biologicals to fluoresce in the red spectrum introduced red artifacts into the images of tissues excited with a 660 nm laser (690 emission filter). However, this limitation does not exclude the possibility that nanoplastics were present at very low levels within the brain. Alternatively, the endogenous clearance pathways may have been sufficient to clear the nanoparticles. It has been shown that zebrafish are capable of clearing nanoparticles from the brain after environmental exposures (Habumugisha et al., 2023). Given the very low exposure rate in the current study, steady-state clearance of nanoplastics may have been sufficient to prevent measurable accumulation. Even if nanoplastics did not accumulate in the brain, peripheral inflammation may have been sufficient to induce elevated microglial immunoreactivity. In fact, gut-derived inflammatory cytokines have been shown to cause neuroinflammation in rats, including microglial activation, after consuming nanoplastics (Yang et al., 2023). Another limitation of the current study is that fish were fed in groups, rather than individually. While it was clear that the fish pursued *Artemia* with equal vigor within each tank, and dominant fish did not prevent other fish from eating the shrimp, our ability to observe the exact volume of *Artemia* consumed by each fish would have been enhanced if fish were fed individually in separate containers.

Future studies will be important for understanding the progression of microglial and astrocytic responses to a nanoplastic-contaminated diet over the course of longer exposures. Additionally, the responses to other types of common nanoplastics besides polystyrene, such as polypropylene and polyethylene will better inform understanding of how current trends in nanoplastic pollution may affect the brains of humans and other animals exposed to nanoplastics through dietary exposures.

## Material and methods

### Test subjects

Prior to experimentation, male and female adult zebrafish (Danio rerio) were purchased from a local supplier (Savannah, GA), and maintained as reported by Mans et al. (2018). All experimental procedures were approved by Georgia Southern University Institutional Animal Use and Care Committee (IACUC).

### Nanoplastics

A stock solution (1% solid) of uniform dyed red fluorescent (660 nm excitation, 690 nm emission; Flash Red) polystyrene nanospheres with a mean diameter of 44 nm was purchased from Bangs Laboratories, Inc (Indiana). Catalog number FSFR001.

### Production of nanoplastic-enriched Artemia for trophic transfer of nanoplastics

Brine shrimp (*Artemia franciscana*) eggs were hatched in 325 mL hatchers over 72 h in 10 parts per thousand (ppt) artificial seawater (ASW) under an environmental chamber ensuring constant illumination with temperatures ranging from 25–28°C. Seventy-two hours was necessary to allow brine shrimp to reach instar II-III stages of development. At this point in development, *Artemia* are no longer attached to the yolk sac and can filter-feed freely. After 72 h post-hatch, the instar II-III nauplii were collected into a sieve and washed under deionized water for 30 s to remove any unhatched eggs or pieces of nauplii. The *Artemia* in the sieve were then suspended in a beaker of ASW before being transferred via a transfer pipette to a glass graduated cylinder for sub-sampling. The volume of shrimp mixture in the glass cylinder was brought to 50 mL using ASW. Prior to sampling, the shrimp mixture was agitated by aspirating and dispensing a large volume of the mixture inside the graduated cylinder using a 100 mL pipette attached to an electric pipette gun. To perform subsampling, 100 microliters of the homogeneous shrimp mixture were transferred into a glass spot place using a micropipette and then divided between another well to generate two, 50 μL subsamples. This procedure was repeated to generate a total of eight, 50 μL subsamples. The brine shrimp were then killed using a single drop of 95% ethanol solution, and each sub-sample was photographed using a stereoscopic dissecting microscope equipped with a digital camera. The number of instar II-III shrimp were counted in each sub-sample using Image-J software (NIH), and the average concentration of nauplii/mL in each subsample was determined to estimate the concentration of nauplii in the bulk solution.

Next, batches of live Artemia were housed in glass beakers containing ASW supplemented with red fluorescent 44 nm polystyrene nanospheres or ASW alone. Enough shrimp were incubated in each beaker to feed groups of four fish at a rate of 1000 shrimp per fish. Therefore, each beaker contained approximately 4000 shrimp. To this end, the appropriate volume of bulk Artemia hatch solution was moved to each glass beaker. The volume of shrimp mixture within each beaker was then raised to 50 mL using ASW. The ASW designated for nanoplastic exposures was supplemented with 15 microliters of nanoplastic (nPS) stock solution (1% w/volume) to produce a final nPS concentration of 0.15 g nPS/50 mL ASW. The Artemia were housed overnight in the ASW solutions with or without nPS, and each beaker received constant gentle aeration via a steel needle to maintain shrimp viability and suspend both the nanoplastics and shrimp homogeneously throughout the aqueous solution.

### Administration of nanoplastic-enriched diet to zebrafish

Fish were housed in groups of four in standard 2.8 L housing tanks (Aquaneering). Each tank contained 2.0 L liters of water and received aeration through an air stone attached to an aquarium pump. Water salinity was maintained at 0.7 millSiemens (mS) conductivity, and the tanks were placed in a temperature-controlled water bath to achieve a stable environmental temperature.

After overnight exposure to nanoplastics, Artemia were collected in a sieve, rinsed with deionized water for 60 s to remove plastic adhering to the outside of the shrimp, suspended in a beaker containing freshwater matching the osmolarity of the zebrafish housing conditions (0.7 mS), and then transferred via a transfer pipette to a 15 mL centrifuge tube. Artemia were still alive after transitioning from ASW to 0.7 mS water, as they can withstand a broad range of salinities. Batches of shrimp were then transported to the housing facility and immediately fed to each tank of zebrafish at a rate of 1000 shrimp per fish. Therefore, each tank received a batch of 4000 shrimp. Fish in the control group received shrimp incubated overnight in ASW without nPS. Zebrafish were monitored during this time to ensure that all fish were eating the Artemia. Zebrafish were fed again at least 2 h later with a nutritionally balanced pellet diet to ensure nutritional needs were met. Shrimp feedings occurred in the morning for fourteen consecutive days. To maintain water quality throughout the experiment, tank water was changed daily prior to Artemia feedings.

### Validation of nPS internalization by Artemia and determination of plastic quantity within shrimp

To visually confirm that the brine shrimp were internalizing nanoplastics, a 50 uL sample of nPS-exposed and control *Artemia* were drop-fixed in 4% paraformaldehyde overnight, fixed to slides, and imaged with a scanning fluorescent confocal microscope using a 660 nm excitation/690 emission filter.

To determine the quantity of nPS internalized by the nPS-exposed brine shrimp, fluorescence spectrophotometry was performed. Four thousand nPS-exposed shrimp or control shrimp were collected in a sieve and rinsed with deionized water for 30 s before being submerged in 95% EtOH to sacrifice the shrimp. Shrimp were then moved to 15 mL Falcon tubes, and volumes were equalized across tubes using a phosphate-buffered saline supplemented with Tween-20 (PBS-T). Samples were concentrated via centrifuge at 3,000 *g* for 10 min at 22°C. Shrimp samples were then moved to a 1.5 mL microcentrifuge tubes, and excess eggs were removed using a micropipette. Shrimp were then homogenized for 60 s using a Teflon-coated pestle in a rotary tool at 10,000 RPM in 0.5 mL PBST homogenization buffer. Homogenized samples were wrapped in aluminum foil and stored in a 4°C refrigerator before fluorescent spectrophotometry. Nanopolystyrene mass standards were prepared in duplicate by supplementing homogenized control shrimp samples with known quantities of fluorescent nPS (0.00 mg, 0.005 mg, 0.010 mg, and 0.040 mg). The fluorescence of nPS standards and experimental samples were measured in a PE Visiplate TC 24-well plate using a fluorescence spectrophotometer (BioTek Synergy H1 Hybrid Reader paired with BioTek Gen5 software) with a 660 excitation/690 emission filter. Results were processed using Microsoft Excel.

### Tissue collection for western blotting and immunostaining

Fish were anesthetized by immersion in tricaine methanesulfonate (300 μg/mL) before decapitation, and the head was stabilized in a foam block submerged in ice-cold artificial cerebrospinal fluid (aCSF) infused with 95% oxygen/5% carbon dioxide. Artificial CSF consisted of (in mM): 120 NaCl, 3.5 KCl, 2.0 CaCl, 1.3 MgSO_4_, 1.3 MgCl_2_, 1.25 NaH_2_PO_4_, 26 NaHCO_3_ and 11 glucose (Mans et al., 2019; Nam et al., 2004). Under guidance of a stereoscopic dissecting microscope tissue was then isolated for western blotting or immunostaining. For western blotting, the telencephalon was isolated and homogenized in Tissue Protein Extraction Reagent (TPER, Thermo Scientific, cat #78510) supplemented with protease inhibitor (Roche cOmplete Mini) and phosphatase inhibitor (Pierce, A32957) cocktails. Samples were homogenized for 60 s using a teflon-coated pestle attached to a rotary tool at 10,000 RPM. Samples were then frozen and stored at −20°C in preparation for western blotting. For immunostaining, entire brains were drop-fixed in 4% paraformaldehyde at room temperature for 2 h then equilibrated overnight in a 30% sucrose solution. Brains were embedded in Tissue-Tek O.C.T. Compound (Sakura Finetek USA Inc, Cat #4583) and cryosectioned at 30 microns through the coronal plane. Sections were mounted on chrome-alum glass slides in sequence. Slides were stored at −20°C.

### Western blotting

Protein concentrations were determined using the Pierce BCA Protein Assay Kit (Thermo Scientific). For blotting 4C4-immunoreactive protein, samples containing 40 μg of total protein were prepared in SDS sample buffer (Invitrogen, Carlsbad, CA, USA) using standard methods. For blotting pAkt and GFAP, samples containing 20 μg of total protein were prepared. Samples were resolved by SDS-PAGE in 10% polyacrylamide gels and blotted to PVDF membranes by semi-dry transfer. Membranes were blocked in 3% milk/TBST for one hr at room temperature prior to application of primary antibody overnight at 4°C. Chemiluminescent protein detection occurred by application of HRP-conjugated secondary antibody for 1 hr at room temperature followed by treatment with Clarity Western ECL (Bio-Rad) peroxidase substrate. Blot luminescence was digitally imaged using a ChemiDoc MP Imaging System with Image Lab Software Version 5.1 (Bio-Rad). Protein levels were quantified by densitometry using ImageJ software (NCBI). A mild or harsh stripping protocol (AbCam) was performed prior to blot re-probing. Beta-actin or glyceraldehyde-3-phosphate dehydrogenase (GAPDH) served as loading controls. Protein levels for 4C4 and GFAP were normalized to loading controls within each blot.

### Antibodies for western blotting

The primary antibodies were rabbit 7.4.C4 (MilliporeSigma 92092321) @ 1:500, rabbit polyclonal GFAP (GeneTex GTX128741) @ 1:1500, rabbit polyclonal GAPDH (Abcam AB210113) @ 1:1000, and rabbit anti-beta-actin (Abcam, ab8227) @ 1:1800. Secondary antibodies were HRP-conjugated anti-rabbit (Cell Signaling, 7074S) @ 1:1000. All antibodies were diluted in 3% milk/TBST.

### 4C4 immunolabeling

Slides were brought to room temperature and rinsed for five minutes in 1% phosphate buffered saline (PBS) three times. After rinsing, slides were incubated in a sheep-donkey blocking solution for one hour at room temperature in a humid chamber. The sheep-donkey blocking solution consisted of 20 mL PBS, 60 microliters 10% bovine serum albumin (VWR Life Science 97061-420), 500 microliters normal donkey serum (Abcam AB7475), 500 microliters normal sheep serum (Abcam AB7489), and 120 microliters Triton X-100 (VWR Life Science 97062-208). To detect microglia, we applied mouse hybridoma 7.4.C4 (MilliporeSigma 92092321) diluted 1:100 in sheep-donkey blocking solution to slides and incubated them overnight in a humid chamber at room temperature. Slides were washed in PBS, then incubated at room temperature with goat anti-mouse AlexaFluor 488 secondary antibody (Invitrogen A-11001) diluted to 1:500 in sheep-donkey blocking solution for 1-h. Slides were again washed in PBS then cover-slipped with DAPI-Fluoromount-G (SouthernBiotech 010020).

### Fluorescent imaging

Slides were imaged using a Zeiss LSM710 Scanning Laser Confocal Microscope paired with Zeiss Zen Black software. The telencephalon was progressively imaged along the ventral to caudal axis to ensure a thorough survey of the telencephalon. Roughly the same number of slices were sampled from every brain with an even distribution of ventral and caudal slices. To sample microglial populations spanning the entire width of each slice, a maximum projection image for each slice was produced using automated tile scans across ten confocal planes spanning 13.5 microns of the *z*-axis (1.5-micron step size). For each tile (708.5 × 708.5-micron scan area), a maximum projection was produced, and tiles were automatically stitched to reconstruct the entire width of each slice. Each scan was repeated at three excitation wavelengths (488 nm, 596 nm, and 660 nm). Three-channel maximum projections were produced from merged images of all three wavelengths in Zen Black. Additionally, a standard background subtraction step was applied equally across every 3-channel maximum projection image within Zen Black. Occasionally, scans included an additional channel of DAPI illumination using a 405 nm laser to identify cell bodies.

### Image analysis

To objectively quantify the density of 4C4-ir microglia within each slice, the color thresholding tool within Image J software was used to quantify the area of green fluorescent microglial cell components occupying each merged 3-channel maximum projection image. Briefly, for each image, the entire slice was outlined as a region of interest (ROI). Next, the total area of the ROI was measured. Green pixels were then selected using the Color Threshold tool and converted to a binary image. The area within the ROI occupied by selected pixels was measured. Finally, the percentage of the ROI occupied by selected pixels was calculated in Microsoft Excel. Summary statistics and graphs were also generated in Excel.

## Data Availability

The raw data supporting the conclusions of this article will be made available by the authors, without undue reservation.
